# Association between Food Insecurity and Sarcopenia among Adults Aged ≥65 Years in Low- and Middle-Income Countries

**DOI:** 10.3390/nu13061879

**Published:** 2021-05-31

**Authors:** Lee Smith, Louis Jacob, Yvonne Barnett, Laurie T. Butler, Jae Il Shin, Guillermo F. López-Sánchez, Pinar Soysal, Nicola Veronese, Josep Maria Haro, Ai Koyanagi

**Affiliations:** 1 The Cambridge Centre for Sport and Exercise Sciences, Anglia Ruskin University, Cambridge CB1 1PT, UK; lee.smith@aru.ac.uk; 2 Research and Development Unit, Parc Sanitari Sant Joan de Déu, CIBERSAM, Dr. Antoni Pujadas, 42, Sant Boi de Llobregat, 08830 Barcelona, Spain; louis.jacob.contacts@gmail.com (L.J.); jmharo@pssjd.org (J.M.H.); 3 Faculty of Medicine, University of Versailles Saint-Quentin-en-Yvelines, 78180 Montigny-le-Bretonneux, France; 4 Faculty of Science and Engineering, Anglia Ruskin University, Cambridge CB1 1PT, UK; yvonne.barnett@aru.ac.uk (Y.B.); laurie.butler@aru.ac.uk (L.T.B.); 5 Department of Pediatrics, Yonsei University College of Medicine, Yonsei-ro 50, Seodaemun-gu, C.P.O. Box 8044, Seoul 120-752, Korea; pedshin2000@gmail.com; 6 Vision and Eye Research Institute, School of Medicine, Faculty of Health, Education, Medicine and Social Care, Anglia Ruskin University, Cambridge CB1 1PT, UK; guillermo.lopez-sanchez@aru.ac.uk; 7 Department of Geriatric Medicine, Bezmialem Vakif University, 34093 Istanbul, Turkey; dr.pinarsoysal@hotmail.com; 8 Department of Geriatric Medicine, University of Palermo, 90133 Palermo, Italy; ilmannato@gmail.com; 9 ICREA, Pg. Lluis Companys 23, 08010 Barcelona, Spain

**Keywords:** food insecurity, sarcopenia, low- and middle-income countries, hunger, old adults

## Abstract

Limited literature has investigated the association between food insecurity and sarcopenia in low- and middle-income countries (LMICs). Therefore, the aim of the present study was to investigate the association between food insecurity and sarcopenia among adults aged ≥65 years in six LMICs. Community-based cross-sectional data of the Study on Global Ageing and Adult Health were analyzed. Sarcopenia was defined as the presence of low skeletal muscle mass based on indirect population formula, and either slow gait or low handgrip strength. In the past, 12-month food insecurity was assessed with two questions on frequency of eating less and hunger due to lack of food. Multivariable logistic regression analysis was conducted. The final sample consisted of 14,585 individuals aged ≥65 years (mean (SD) age 72.6 (11.5) years; 55.0% females). The prevalence of sarcopenia among those with no food insecurity was 13.0% but this increased to 24.4% among those with severe food insecurity. After adjustment for potential confounders, compared to no food insecurity, severe food insecurity was associated with 2.05 (95%CI = 1.12–3.73) times higher odds for sarcopenia. In this large representative sample of older adults from multiple LMICs, it was found that severe food insecurity is associated with higher odds for sarcopenia. Addressing food insecurity in such settings may be an effective strategy to curb the high prevalence of sarcopenia in LMICs.

## 1. Introduction

Sarcopenia is defined as “age-related muscle loss, affecting a combination of appendicular muscle mass, muscle strength, and/or physical performance measures” [1]. In September 2016, sarcopenia was included in the ICD-10-CM as a medical condition [2]. Literature has revealed that the global prevalence of sarcopenia is high in older adults (between 5 to 10%), with a particularly high prevalence in low- and middle-incomes countries (LMICs) [3,4]. For example, one study in older Gambian men and women showed a prevalence of up to 20% and 45%, respectively [5]. Such a high prevalence of sarcopenia is of concern as sarcopenia has been associated with multiple detrimental health outcomes. In a recent umbrella review, sarcopenia was associated with several adverse health-related outcomes in older people, including mortality and disability [6]. Therefore, there is an urgent need to investigate the risk factors for sarcopenia so that effective interventions can be implemented. It is also important to note that when increased amounts of adipose tissue accompany sarcopenia, it is referred to as sarcopenic obesity [7]. Sarcopenic obesity can increase risk above and beyond that of sarcopenia or obesity alone in terms of multiple non-communicable diseases, and long-term disability [8].

Previously reported risk factors for sarcopenia include low levels of physical activity, cardio-respiratory fitness and strength, and high levels of sedentary time [9]. Furthermore, nutrition has also been reported to be important in the development of sarcopenia. For example, a diet high in protein as well as high fruit consumption and a Mediterranean diet have been associated with lower risk for sarcopenia [10,11,12]. Furthermore, the lack of some micronutrients, particularly antioxidant micronutrients, have been implicated in the etiology of sarcopenia [13]. 

However, there is limited research on the association between food insecurity and sarcopenia. Food insecurity may be defined as the disruption of food intake or eating patterns because of lack of money and other resources [14] and is highly prevalent, especially in LMICs [15]. Food insecurity can lead to poor nutrition as food insecurity often compromises diet quality, as people tend to switch to more affordable but less nutritious food when food is scarce (e.g., high fat and carbohydrates, low vitamins, and micronutrients) [16]. Poor nutrition, in turn, may increase risk for sarcopenia [13]. 

There is currently a small but growing body of literature investigating the relationship between food insecurity and sarcopenia. One study carried out in the US including 2965 subjects aged ≥60 years concluded that food insecurity was strongly associated with sarcopenia [17]. However, another study carried out in one LMIC (Mexico) and utilizing a small sample (*n* = 168) of older adults aged ≥60 years observed a lack of association between food insecurity and sarcopenia [18]. Other research in LMICs have found that older individuals experiencing food insecurity have a lower mean standard deviation of muscle mass strength and physical performance, which are proxy measures for sarcopenia [19]. Clearly, more research is required to elucidate the association between food insecurity and sarcopenia in large representative samples of older adults from LMICs. 

Given this background, the aim of the present study was to investigate the association between food insecurity and sarcopenia in a large nationally representative sample of adults aged ≥65 years from six LMICs. It is particularly important to study this association in LMICs as there is a high level of food insecurity and sarcopenia in these settings [5,20]. 

## 2. Methods

For the present analyses, data from SAGE were utilized and this data can be publicly accessed through the following link: http://www.who.int/healthinfo/sage/en/ (accessed on 30 May 2021). This survey was administered between 2007 and 2010 in the following countries: China, Ghana, India, Mexico, Russia, and South Africa. All countries were LMICs based on the World Bank classification at the time of the survey. Details of the survey methodology have been published elsewhere [21]. In brief, in order to obtain nationally representative samples, a multistage clustered sampling design method was used. The sample consisted of adults aged ≥18 years with oversampling of those aged ≥50 years. Trained interviewers conducted face-to-face interviews using a standard questionnaire. Standard translation procedures were undertaken to ensure comparability between countries. The survey response rates were: China 93%; Ghana 81%; India 68%; Mexico 53%; Russia 83%; and South Africa 75%. Sampling weights were constructed to adjust for the population structure as reported by the United Nations Statistical Division. Ethical approval was obtained from the WHO Ethical Review Committee and local ethics research review boards. Written informed consent was obtained from all participants.

### 2.1. Sarcopenia

Following the criteria used in previous publications using the same dataset [22,23], sarcopenia was defined as having low skeletal muscle mass (SMM) as reflected by lower skeletal mass index (SMI) and either a slow gait speed or a weak handgrip strength [24]. Skeletal muscle mass (SMM) was calculated as the appendicular skeletal muscle mass (ASM) based on the equation proposed by Lee and colleagues: ASM = 0.244*weight + 7.8*height + 6.6*sex–0.098*age + race–3.3 (where female = 0 and male = 1; race = 0 (White and Hispanic), race = 1.9 (Black), and race = −1.6 (Asian)) [25]. ASM was further divided by BMI based on measured weight and height to create a skeletal muscle mass index (SMI) [26]. Low SMM was defined as the lowest quintile of the SMI based on sex-stratified values. Country-specific cut-offs were only used to determine low SMI, as this indicator is likely to be affected by racial differences in body composition [27]. Gait speed was based on a 4 m timed walk and was measured by asking the participant to walk at a normal pace. The interviewer recorded the time to completion of the 4 m walk. Slow gait speed referred to the lowest quintile of walking speed based on height, age, and sex-stratified values [28,29]. Weak handgrip strength was defined as <27 kg for men and <16 kg for women using the average value of the two handgrip measurements of the dominant hand [30].

### 2.2. Food Insecurity

Food insecurity was defined with the use of the two following questions: “In the last 12 months, how often did you ever eat less than you felt you should because there wasn’t enough food?” and “In the last 12 months, were you ever hungry, but didn’t eat because you couldn’t afford enough food?” Both of these questions had as answer options: every month (coded = 1); almost every month (coded = 2); some months, but not every month (coded = 3); only in 1 or 2 months (coded = 4); never (coded = 5). These items were adapted from similar items in food security questionnaires such as the US Household Food Security Survey Module and National Health and Nutrition Examination Survey (NHANES) Food Security module. As in previous SAGE studies, those who answered 1 through 3 to both questions or answered 1 to either question were coded as severely food insecure. Those who did not meet the criteria for severe food insecurity but answered 2 through 4 for either question were coded as moderately food insecure. Those who answered 5 to both questions were food secure [31,32]. 

### 2.3. Control Variables

The control variables were selected based on previous literature [10] and included sex, age, wealth, years of education received, alcohol consumption in the past 30 days (yes or no), smoking (never, current, past), physical activity, body mass index (BMI) based on measured weight and height (<18.5 kg/m^2^ (underweight), 18.5–24.9 kg/m^2^ (normal weight), 25.0–29.9 kg/m^2^ (overweight), ≥30 kg/m^2^ (obese)), and number of chronic diseases. Levels of physical activity were assessed with the Global Physical Activity Questionnaire and were classified as low, moderate, and high based on conventional cut-offs [33]. The total number of 11 chronic physical conditions (angina, arthritis, asthma, stroke, diabetes, edentulism, visual impairment, chronic lung disease, hypertension, chronic back pain, and hearing problems) was calculated for each participant. The diagnosis was based on the presence of either one of the following: self-reported diagnosis, or symptom-based diagnosis based on algorithms, etc. 

### 2.4. Statistical Analysis

The statistical analysis was performed with Stata 14.1 (Stata Corp LP, College Station, TX, USA). The analysis was restricted to those aged ≥65 years, as sarcopenia is an age-related condition. The difference in sample characteristics by food insecurity status was tested by Chi-squared tests and one-way ANOVA for categorical and continuous variables, respectively. We conducted multivariable logistic regression analysis with food insecurity as the exposure variable and sarcopenia as the outcome. In order to assess the influence of different variables in the association between food insecurity and sarcopenia, we constructed four models: Model 1—adjusted for sociodemographic variables (sex, age, wealth, education, country); Model 2—adjusted for factors in Model 1 and behavioral factors (alcohol consumption, smoking, physical activity); Model 3—adjusted for factors in Model 2 and BMI; Model 4—adjusted for factors in Model 3 and number of chronic diseases (fully adjusted model). We also assessed whether there is effect modification by sex in the association between food insecurity and sarcopenia by including an interaction term (food insecurity X sex) in the fully adjusted model. Dummy variables for each country were included in the model to adjust for country [32,33]. All variables were included in the models as categorical variables with the exception of age, education, and number of chronic diseases (continuous variables). The sample weighting and the complex study design were taken into account in the analyses to generate nationally representative estimates. Results from the regression analyses are presented as odds ratios (ORs) with 95% confidence intervals (CIs). The level of statistical significance was set at *p* < 0.05. 

## 3. Results

**Figure 1:**
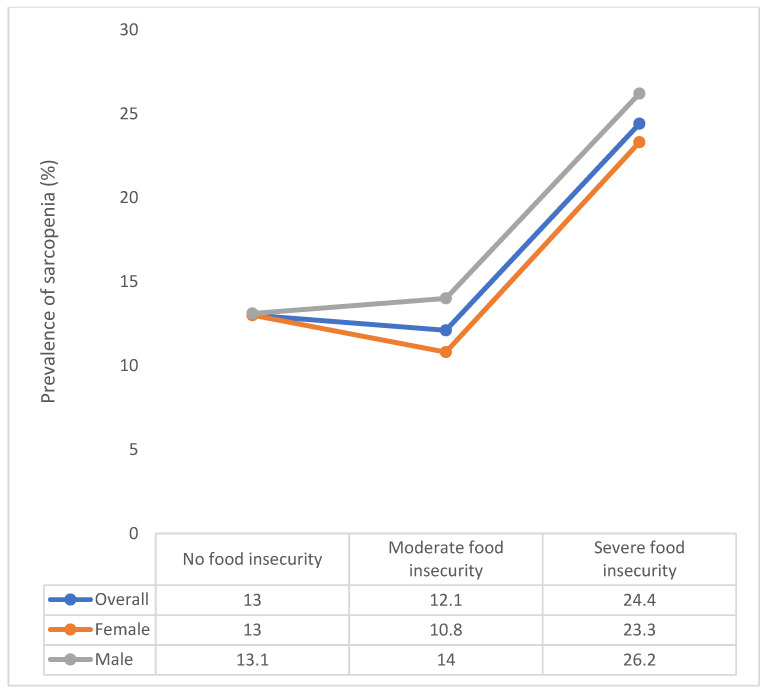
Prevalence of sarcopenia by food insecurity status (overall and by sex).

The final sample consisted of 14,585 individuals (China *n* = 5360; Ghana *n* = 1975; India *n* = 2441; Mexico *n* = 1375; Russia *n* = 1950; South Africa *n* = 1484) aged ≥65 years with a mean (SD) age of 72.6 (11.5) years and 55.0% were females. The prevalence of sarcopenia was 13.5%, while that of moderate and severe food insecurity were 6.7% and 5.0%, respectively. The sample characteristics are shown in Table 1. Food insecurity was more common among females, the poorer, and those with less education. The prevalence of sarcopenia was particularly high among those with severe food insecurity in the overall sample as well as those stratified by sex (Figure 1). For example, in the overall sample, the prevalence of sarcopenia among those with no food insecurity was 13.0% but this increased to 24.4% among those with severe food insecurity. The association between food insecurity and sarcopenia estimated by multivariable logistic regression is shown in Table 2. In the fully adjusted model (Model 4), compared to no food insecurity, severe food insecurity was associated with 2.05 (95%CI = 1.12–3.73) times higher odds for sarcopenia. The sequential inclusion of different variables in the model had almost no influence in the association between food insecurity and sarcopenia (Model 1 to 4). Interaction analysis showed that there is no effect modification in the association between food insecurity and sarcopenia by sex. 

## 4. Discussion

### 4.1. Main Findings and Comparison with Previous Literature

In this large representative sample of older adults from six LMICs, it was observed that compared to no food insecurity, severe food insecurity was associated with 2.05 times higher odds for sarcopenia. Our findings support literature that has demonstrated an association between food insecurity and a higher risk of sarcopenia [17] and adds to this by demonstrating such an association in a large representative sample of older adults from six LMICs, which collectively represents nearly half of the worldwide population. The present findings also support literature investigating food insecurity with a more proxy outcome (muscle mass strength and physical performance) [19]. However, the present findings contradict the only other existing study on this topic carried out in a LMIC (Mexico) that observed no significant associations [18]. However, this study only included 168 participants and it is possible that it lacked statistical power to detect a difference.

### 4.2. Interpretation of Findings

Interestingly, the present analysis demonstrated that the control variables included in this study (e.g., physical activity, BMI, chronic physical diseases) have very little influence in the association between food insecurity and sarcopenia. Therefore, this association may predominantly be explained by factors which were not included in our study such as nutritional factors. Specifically, food insecurity is associated with multiple micro- and macro-nutrient deficiencies, many of which deficiencies may increase risk of sarcopenia. For example, exogenous antioxidant vitamins contribute to the maintenance of skeletal muscle mass. Importantly, in the ageing population endogenous antioxidant efficiency is reduced [34,35,36,37]. Indeed, a higher dietary intake of antioxidants (e.g., vitamin C and beta-carotene) has been shown to be associated with higher skeletal muscular strength among older adults residing in Italy [38]. Next, food insecurity has been found to be associated with a suboptimal intake of folate [39], and low folate intake has been found to be associated with increased risk of sarcopenia [40]. 

Finally, food insecurity, particularly hunger, may lead to energy deficiency. Acute energy deficiency is associated with changes in body composition, as well as a reduced basal metabolic rate, and thus likely increases risk of sarcopenia [41]. 

### 4.3. Public Health Implications and Areas for Future Research

The results of our study show that interventions to combat food insecurity among older people in LMICs may also aid in the prevention of sarcopenia. One such intervention may include the implementation of food banks which have been highly successful in high-income countries to tackle food insecurity [42]. Another successful intervention in high-income countries specifically targeted at older adults is home delivery of meals supported by governments [43,44]. However, such initiatives are rare in LMICs and will require strong governmental “buy in” to implement. In addition, future studies should assess the content of food that food insecure older people tend to consume in LMICs, and how quantity and diversity of food consumed in this population influences health outcomes, including sarcopenia. 

### 4.4. Strengths and Limitations

The large sample of older adults from multiple LMICs is a clear strength of the present work. However, findings must be considered in light of the study’s limitations. First, the study is cross-sectional in nature and thus the direction of the association cannot be confirmed. However, it is unlikely that sarcopenia drives food insecurity rather than vice versa. Second, the study relied mostly on self-reported data which could have been affected by factors such as recall and social desirability biases. Finally, the measure of food insecurity used in our study was based on two questions and did not constitute a comprehensive food insecurity measure. 

## 5. Conclusions

In this large representative sample of older adults from multiple LMICs, it was found that severe food insecurity is associated with higher odds for sarcopenia. Addressing food insecurity in such settings may be an effective strategy to curb the high prevalence of sarcopenia in LMICs. 

## Figures and Tables

**Table 1:** Sample characteristics (overall and by food insecurity).

			Food Insecurity			
Characteristic		Overall	None	Moderate	Severe	*p*-Value ^a^
Sex	Male	45.0	46.1	37.7	37.8	0.009
	Female	55.0	53.9	62.3	62.2	
Age (years)	Mean (SD)	72.6 (11.5)	72.5 (11.0)	71.9 (12.6)	72.5 (14.9)	0.177
Wealth	Poorest	21.7	19.5	35.3	41.6	<0.001
	Poorer	21.0	20.0	29.3	26.9	
	Middle	20.4	20.4	22.3	18.6	
	Richer	17.5	18.8	8.0	7.6	
	Richest	19.4	21.4	5.1	5.3	
Education (years)	Mean (SD)	5.2 (9.3)	5.4 (9.1)	3.9 (9.7)	3.6 (10.7)	<0.001
Alcohol consumption	No	86.1	85.9	87.2	87.1	0.755
	Yes	13.9	14.1	12.8	12.9	
Smoking	Never	62.2	62.9	57.3	56.8	0.139
	Current	29.3	28.6	33.2	35.7	
	Quit	8.5	8.5	9.5	7.5	
Physical activity	High	35.2	34.0	45.1	43.7	<0.001
	Moderate	25.2	26.3	17.8	15.1	
	Low	39.6	39.7	37.1	41.2	
BMI (kg/m^2^)	<18.5	19.3	17.5	32.4	34.8	<0.001
	18.5–24.9	46.4	47.3	42.6	35.5	
	25.0–29.9	23.9	25.1	14.7	14.9	
	≥30	10.4	10.2	10.3	14.8	
No. of chronic diseases	Mean (SD)	2.1 (2.8)	2.1 (2.7)	2.4 (3.7)	2.6 (4.1)	<0.001

Abbreviation: SD Standard deviation; BMI Body mass index; ^a^ *p*-value was obtained by Chi-squared test and one-way ANOVA for categorical and continuous variables, respectively.

**Table 2:** Association between food insecurity and sarcopenia (outcome) estimated by multivariable logistic regression.

		Model 1		Model 2		Model 3		Model 4	
Characteristic		OR	95%CI	OR	95%CI	OR	95%CI	OR	95%CI
Food insecurity	None	1.00		1.00		1.00		1.00	
	Moderate	0.82	[0.56,1.21]	0.84	[0.57,1.25]	0.87	[0.59,1.28]	0.87	[0.59,1.30]
	Severe	1.98 *	[1.10,3.58]	2.04 *	[1.13,3.68]	2.11 *	[1.16,3.85]	2.05 *	[1.12,3.73]
Sex	Male	1.00		1.00		1.00		1.00	
	Female	0.81 *	[0.66,0.99]	0.77 *	[0.60,0.99]	0.72 *	[0.56,0.93]	0.72 **	[0.56,0.92]
Age (years)		1.12 ***	[1.10,1.14]	1.11 ***	[1.10,1.13]	1.12 ***	[1.10,1.14]	1.12 ***	[1.10,1.14]
Wealth	Poorest	1.00		1.00		1.00		1.00	
	Poorer	0.76	[0.56,1.04]	0.77	[0.56,1.05]	0.73	[0.53,1.01]	0.76	[0.55,1.04]
	Middle	0.78	[0.55,1.10]	0.79	[0.55,1.11]	0.75	[0.52,1.07]	0.74	[0.51,1.06]
	Richer	0.66 **	[0.49,0.89]	0.65 **	[0.48,0.87]	0.60 **	[0.44,0.82]	0.60 **	[0.44,0.82]
	Richest	0.54 **	[0.35,0.81]	0.53 **	[0.35,0.80]	0.45 ***	[0.28,0.71]	0.46 **	[0.29,0.73]
Education (years)		0.95 ***	[0.92,0.98]	0.95 ***	[0.92,0.98]	0.94 ***	[0.92,0.97]	0.95 ***	[0.92,0.98]
Alcohol consumption	No			1.00		1.00		1.00	
	Yes			0.74	[0.52,1.04]	0.75	[0.53,1.06]	0.75	[0.53,1.08]
Smoking	Never			1.00		1.00		1.00	
	Current			0.98	[0.74,1.30]	1.04	[0.78,1.39]	1.02	[0.76,1.37]
	Quit			1.19	[0.83,1.69]	1.18	[0.83,1.68]	1.12	[0.78,1.60]
Physical activity	High			1.00		1.00		1.00	
	Moderate			1.23	[0.97,1.55]	1.21	[0.95,1.54]	1.14	[0.89,1.45]
	Low			1.22	[0.97,1.53]	1.20	[0.95,1.52]	1.12	[0.89,1.42]
BMI (kg/m^2^)	18.5–24.9					1.00		1.00	
	25.0–29.9					1.36 **	[1.08,1.70]	1.36 **	[1.09,1.70]
	≥30					2.60 ***	[1.65,4.10]	2.48 ***	[1.55,3.97]
	<18.5					0.63 **	[0.44,0.89]	0.64 *	[0.45,0.92]
No. of chronic diseases								1.14 ***	[1.06,1.22]

Abbreviation: OR Odds ratio; CI Confidence interval; BMI Body mass index. Models are adjusted for all variables in the respective column and country. * *p* < 0.05, ** *p* < 0.01, *** *p* < 0.001.

## Data Availability

The data presented in this study are available on request from the corresponding author.
